# Assessing functional properties of diet protein hydrolysate and oil from fish waste on canine immune parameters, cardiac biomarkers, and fecal microbiota

**DOI:** 10.3389/fvets.2024.1449141

**Published:** 2024-11-11

**Authors:** Ana R. J. Cabrita, Carolina Barroso, Ana Patrícia Fontes-Sousa, Alexandra Correia, Luzia Teixeira, Margarida R. G. Maia, Manuel Vilanova, Timur Yergaliyev, Amélia Camarinha-Silva, António J. M. Fonseca

**Affiliations:** ^1^REQUIMTE, Network of Chemistry and Technology, LAQV, Laboratory for Green Chemistry, ICBAS, School of Medicine and Biomedical Sciences, University of Porto, Porto, Portugal; ^2^Department of Immuno-Physiology and Pharmacology, Center for Pharmacological Research and Drug Innovation (MedInUP), ICBAS, School of Medicine and Biomedical Sciences, Veterinary Hospital of the University of Porto (UPVET), University of Porto, Porto, Portugal; ^3^ICBAS – School of Medicine and Biomedical Sciences, University of Porto, Porto, Portugal; ^4^i3S – Instituto de Investigação e Inovação em Saúde, University of Porto, Porto, Portugal; ^5^HoLMiR – Hohenheim Center for Livestock Microbiome Research, University of Hohenheim, Stuttgart, Germany; ^6^Institute of Animal Science, University of Hohenheim, Stuttgart, Germany

**Keywords:** cardiac evaluation, fecal microbiota, fish waste, functionality, immune response, pet food

## Abstract

Locally produced fish hydrolysate and oil from the agrifood sector comprises a sustainable solution both to the problem of fish waste disposal and to the petfood sector with potential benefits for the animal’s health. This study evaluated the effects of the dietary replacement of mainly imported shrimp hydrolysate (5%) and salmon oil (3%; control diet) with locally produced fish hydrolysate (5%) and oil (3.2%) obtained from fish waste (experimental diet) on systemic inflammation markers, adipokines levels, cardiac function and fecal microbiota of adult dogs. Samples and measurements were taken from a feeding trial conducted according to a crossover design with two diets (control and experimental diets), six adult Beagle dogs per diet and two periods of 6 weeks each. The experimental diet, with higher docosahexaenoic (DHA) and eicosapentaenoic (EPA) acids contents, decreased plasmatic triglycerides and the activity of angiotensin converting enzyme, also tending to decrease total cholesterol. No effects of diet were observed on serum levels of the pro-inflammatory cytokines interleukin (IL)-1β, IL-8, and IL-12/IL-23 p40, and of the serum levels of the anti-inflammatory adipokine adiponectin. Blood pressure, heart rate and echocardiographic measurements were similar between diets with the only exception of left atrial to aorta diameter ratio that was higher in dogs fed the experimental diet, but without clinical relevance. Diet did not significantly affect fecal immunoglobulin A concentration. Regarding fecal microbiome, *Megasphaera* was the most abundant genus, followed by *Bifidobacterium*, *Fusobacterium*, and *Prevotella*, being the relative abundances of *Fusobacterium* and *Ileibacterium* genera positively affected by the experimental diet. Overall, results from the performed short term trial suggest that shrimp hydrolysate and salmon oil can be replaced by protein hydrolysate and oil from fish by-products without affecting systemic inflammatory markers, cardiac structure and function, but potentially benefiting bacterial genera associated with healthy microbiome. Considering the high DHA and EPA contents and the antioxidant properties of fish oil and hydrolysate, it would be worthwhile in the future to assess their long-term effects on inflammatory markers and their role in spontaneous canine cardiac diseases and to perform metabolomic and metagenomics analysis to elucidate the relevance of microbiota changes in the gut.

## 1 Introduction

In recent years, several studies have explored fish by-products to produce various high-value-added products such as protein concentrates, bioactive peptides, fish oils, among others (1). These products can be used as ingredients in the production of food intended for human consumption, however the development of petfood formulations has emerged as a very promising and economically viable solution. Indeed, these protein and lipid sources are an excellent option for enriching petfood in amino acids and essential fatty acids with the advantage of the absence of anti-nutritional factors or allergenic proteins and potential benefits for the animal’s health (2, 3). The main bioactivities of fish hydrolysates reported in the literature include antioxidant, antihypertensive, anti-obesity, anti-inflammatory, anticancer, antimicrobial and neuroprotective activities (4, 5).

Fish oil can be highlighted by the long-chain polyunsaturated fatty acids (PUFA) content, namely eicosapentaenoic acid (EPA, 20:5 *n*-3) and docosahexaenoic acid (DHA, 22:6 *n*-3). These long-chain omega (*n*)-3 PUFA are precursors of several metabolites that act as lipid mediators and are beneficial in the prevention of certain diseases, being used in therapeutic diets (e.g., cardiovascular, renal, gastrointestinal, orthopedic and dermatological) at high doses (6–10).

In addition to the economic, nutritional and bioactivity benefits associated with the valorization of fish by-products in the petfood sector, this also makes it possible to increase the environmental sustainability of the sector. In fact, the growing number of companion animals has raised concerns about their sustainability, and the pet food industry is starting to tackle this issue particularly through finding alternative protein and energy food resources (11–13).

A recent study (14) showed that the dietary inclusion of fish hydrolysate and oil locally obtained from fish waste in substitution of shrimp hydrolysate and salmon oil mainly imported from third countries was well accepted by dogs, not affecting food intake, digestibility, or fecal characteristics, but promoting blood EPA, DHA, and omega-3 index, suggesting a potential health-promoting effect. To further gain insight into the potential functional role of these upcycled resources, the present study used samples and measurements collected during the earlier experiment (14) to evaluate effects on health parameters, including immune effector molecules, fecal microbiota, and cardiac function.

## 2 Materials and methods

The trial was approved by the Animal Ethics Committee of School of Medicine and Biomedical Sciences, University of Porto, licensed by the Portuguese General Directorate of Food and Veterinary Medicine (Permit N° 0421/000/000/2021), and conducted by trained scientists in laboratory animal science (FELASA, category C) in line with good animal welfare practices (European Union Directive 2010/63/EU).

### 2.1 Animals, diets, and experimental design

Details on animals, diets and experimental design of the trial were earlier reported (14). Briefly, 12 healthy adult Beagle dogs (2 intact males, 4 neutered males, and 6 spayed females), 5.4 ± 0.57 years old, weighing 11.8 ± 2.20 kg with a body condition score of 4.3 ± 0.69 (assessed according to a 9 point-scale; 15), and housed at the kennel of the School of Medicine and Biomedical Sciences, University of Porto, were used. A commercial diet for adult medium-size dogs (Sorgal Pet Food, Ovar., Portugal) with the inclusion of 5% shrimp hydrolysate (Symrise Aqua, Equator) and 3% salmon oil (Symrise Aqua, Norway) was used as the control diet. The experimental diet comprised the same ingredients of the control diet, with slight adjustments on wheat grain (6.0 and 7.5% for the control and experimental diet), pea concentrate (7.0 and 5.0%), and poultry fat (5.0 and 5.3%), and the replacement of 5% shrimp hydrolysate with 5% fish hydrolysate, and 3% of salmon oil with 3.2% of fish oil. Fish hydrolysate was obtained by enzymatic hydrolysis with the non-specific serine endopeptidase from *Bacillus licheniformis* Alcalase 2.4 L (Novozymes^®^, Bagsvaerd, Denmark) and fish oil were obtained from fish by-products comprising heads, tails, skin, slices, and whole fish mainly of salmon, sea bream, sea bass and red fish, and provided by a company group (Empresa Transformadora de Subprodutos Animais S.A., ETSA, Loures, Portugal) dedicated to recycling in the food sector.

A detailed characterization of both diets was earlier presented (14). Briefly, comparing to the control diet, the experimental diet presented lower crude protein (27.0 vs. 29.1 g 100 g^−1^ dry matter, DM), but similar amino acids profile, and higher ether extract (11.6 vs. 10.4 g 100 g^−1^ DM) contents with a higher percentage of *n*-3 PUFA (6.44 vs. 3.48 g 100 g^−1^ total fatty acids), particularly EPA (1.25 vs. 0.355 g 100 g^−1^ total fatty acids), docosapentaenoic acid (C22:5 *n*-3; 0.360 vs. 0.116 g 100 g^−1^ total fatty acids), and DHA (1.96 vs. 0.494 g 100 g^−1^ total fatty acids), and a lower percentage of *n*-6 PUFA (22.1 vs. 27.0 g 100 g^−1^ total fatty acids).

Before the start of the trial, the dogs were considered clinically healthy based on a comprehensive examination conducted by a veterinarian. Additionally, given the age and breed of the dogs, a baseline echocardiogram was performed, revealing thickened mitral valve leaflets in all cases, consistent with myxomatous mitral valve disease. Mild mitral valve regurgitation was observed in seven dogs, all of which showed no signs of cardiac remodeling (stage B1) (16). The severity of mitral regurgitation was assessed semiquantitatively by measuring the ratio of the mitral regurgitant maximal jet area to the left atrial area by way of color Doppler. A result of <5% was considered trace, between 5 and 20% was considered mild, between 20 and 50% was considered moderate, and >50% was considered severe (17, 18). Dogs were divided into two groups blocked for sex and received the control and experimental diets in two consecutive experimental periods of 6 weeks each following a crossover arrangement. Animals were individually fed twice a day (8:30 h and 17:00 h) with the daily ration calculated according to body condition score (15) and the ideal body weight (BW) to meet the metabolizable energy requirements (19).

### 2.2 Blood collection and analysis

Blood samples were collected from each dog in 1 day of the last week of each experimental period before the morning meal, from the jugular vein into EDTA BD Vacutainer (VWR International, Carnaxide, Portugal) and VACUETTE BD Vacutainer^®^ SST™ II tubes with clot activator and separating gel (Becton, Dickinson and Company, Franklin Lakes, NJ, USA). Blood was allowed to clot for 60 min at room temperature before centrifugation at 1200⨯g for 10 min. Serum was collected, divided into aliquots, and stored at −80°C until analysis. Insulin, cholesterol LDL, cholesterol HDL, aldosterone, insulin-like growth factor (IGF-1), cytokines, leptin and adiponectin were tested in serum, whereas the other parameters analyzed were measured in EDTA plasma. A mid-volume analyzer (Cobas c501, Roche Diagnostics, Indianapolis, IN, USA) were used to analyze total protein, albumin, glucose, creatinine, urea, alanine aminotransferase, alkaline phosphatase, total cholesterol, triglycerides, and reactive C protein. Globulin concentration was determined as the difference between total protein and albumin. Troponin I, IGF-1 (Siemens Immulite 2000, Siemens Healthineers, Erlanger, Germany), insulin (Siemens Advia Centaur, Siemens Healthineers) and angiotensin II (Snibe Maglumi, Shenzhen, China) were analyzed by chemiluminescence immunoassays. Cholesterol LDL and HDL, and angiotensin converting enzyme activity were determined through molecular absorption spectrometry (Siemens Atellica, Siemens Healthineers). NT-pro-BNP was quantified by ELISA using the canine cardiopet proBNP assay (IDEXX Laboratories, Westbrook, ME, USA). Aldosterone and plasmatic renin activity were measured through radioimmunoassays (Gamma WIZARD 2470 Perkin Elmer, Waltham, MA, USA).

To evaluate the effect of diet on basal systemic inflammation, the concentrations of the pro-inflammatory cytokines tumor necrosis factor (TNF)-*α*, interleukin (IL)-1β, IL-8, IL-12/IL-23 p40, and interferon (IFN)-*γ*, and of the anti-inflammatory cytokine IL-10 were quantified in the serum using the following ELISA kits, according to the manufacturers’ instructions: IL-1β (DY3747; Canine DuoSet ELISA, R&D Systems, Oxford, UK), IL-10 (DY735; Canine DuoSet ELISA, R&D Systems), IL-12/IL-23 p40 (DY1969; Canine DuoSet ELISA, R&D Systems), TNF-*α* (DY1507, Canine DuoSet ELISA, R&D Systems), IFN-*γ* (DY781B, Canine DuoSet ELISA, R&D Systems), and IL-8 (3114-1H-20, ELISA Flex Bovine IL-8, Mabtech AB, Nacka, Sweden). The colorimetric detection was performed using a Multiskan EX microplate reader (Thermo Fisher Scientific, Carlsbad, MA, USA) using the Ascent software (Thermo Fisher Scientific). ELISA kits specific to canine adipokines were also used to analyse serum concentrations of leptin (Canine Leptin Cat. No. EZCL-31 K, Merck Millipore, Darmstadt, Germany) and adiponectin (Canine Adiponectin ELISA Kit, Cat. No. RD-ADP-c, Reddot Biotech Inc., Kelowna, Canada), following manufacturers’ instructions. For adiponectin quantification, serum samples were previously diluted 1:1000 in phosphate buffered saline (PBS) medium to be within the range of the standard curve (100–1.562 ng mL^−1^). For leptin quantification, 20 μL of serum samples were applied to each plate well containing 80 μL of assay buffer, as recommended by the manufacturer. A Multiskan™ FC Microplate Photometer (Thermo Fisher Scientific) with the SkanIt Software 3.1 was used to measure absorbance at 450 nm and to determine sample concentration through the four-parameter logistic curve.

### 2.3 Blood pressure, echocardiogram, and electrocardiogram

Measurements of blood pressure, echocardiogram and electrocardiogram were conducted on each dog on 1 day of the last week of each experimental period. All procedures were performed while the dogs were conscious and without sedation. The assessment was carried out and analyzed by a single researcher. Systolic, diastolic, and mean arterial pressures were measured using a veterinary-specific oscillometric non-invasive blood pressure system (Vet20 SunTech; SunTech Medical Inc., Morrisville, NC, USA), in accordance with the American College of Veterinary Internal Medicine (ACVIM) Consensus guidelines (20). Five readings per dog were averaged to determine the blood pressure values.

After blood pressure measurements were obtained, the dogs underwent shaving to create a window for echocardiography. Conventional echocardiography (2D, M-mode, color Doppler and spectral Doppler), with a single-lead simultaneous electrocardiogram, was conducted using an M5S phased-array transducer (1.5–4.5 MHz) and a GE Logiq S8 XDclear echocardiograph (GE HealthCare, Chicago, IL, USA), following a 15-min resting period. Data were digitally stored for subsequent measurements, adhering to the guidelines of the Echocardiography Committee of the Specialty of Cardiology of ACVIM (21). Acoustic gel was applied over the transducer and directly onto the clipped skin. To minimize variability, a single trained operator recorded three consecutive measurements for each parameter, as previously recommended (22).

For the right parasternal views, the dogs were positioned in right lateral recumbency. M-mode imaging was applied on the right parasternal short-axis view at the level of the left ventricular papillary muscles to assess the interventricular septum (IVS), left ventricle (LV) internal diameter (LVID), and LV posterior wall (LVPW) in diastole (d) and systole (s). The LVIDd was normalized to BW using the formula (23):


$$\text{LVIDd normalized}\;\left(\text{LVIDdN}\right)=\frac{\text{LVIDd}\;\left(cm\right)}{BW\;{\left(kg\right)}^{0.294}}$$

Fractional shortening (FS) and ejection fraction (EF) of the LV were calculated as follows (24):


$$FS\;\left(\%\right)=\frac{\text{LVIDd}-\text{LVIDs}}{\text{LVIDd}}\times 100$$

$$EF\;\left(\%\right)=\frac{{\text{LVIDd}}^{3}-{\text{LVIDs}}^{3}}{{\text{LVIDd}}^{3}}\times 100$$

The right parasternal long-axis view with 2D-guided M-mode was utilized to measure the E-point-to-septal separation interval (EPSS) in the plane of the mitral valves.

The diameters of the left atrium (LA) and aorta (Ao) were measured from the right parasternal short-axis view at the level of the Ao aligned with the commissure of the aortic non-coronary and left coronary cusps during early ventricular diastole, following established protocols which exclude the pulmonary veins (25). The LA to Ao ratio (LA/Ao) was calculated and documented.

Pulmonary flow velocities were assessed using pulsed-wave Doppler imaging from the right parasternal short-axis view. Aortic and mitral flows were evaluated with pulsed-wave Doppler from the left parasternal apical 5- and 4-chamber views, respectively. For the transmitral flow, the sample volume was positioned just above the mitral valve leaflets during diastole, and peak flow velocities during early diastole (E-wave) and atrial contraction (A-wave) were measured.

Heart rate (HR) was directly calculated from the inter-beat intervals of the electrocardiographic tracing. Stroke volume (SV) was derived as the difference between EDV and ESV, where EDV represents the end diastolic volume calculated by the formula:


$$EDV=\frac{7\times \frac{\text{LVDd}}{10^{3}}}{2.4+\frac{\text{LVDd}}{10}}$$

and ESV denotes the end systolic volume, calculated by:


$$ESV=\frac{7\times \frac{\text{LVDs}}{10^{3}}}{2.4+\frac{\text{LVDs}}{10}}$$

Cardiac output (CO) was determined as the product of SV and HR (24).

### 2.4 Fecal immunoglobulin A concentration and microbiota

In 5 days of the last week of each experimental period, total feces excreted by dogs were collected. Individual samples were weighed, mixed, subsampled at different locations and immediately frozen at −20°C throughout the collection for later analysis of immunoglobulin A (IgA) concentration and fecal microbiome. Immediately before performing these analyses, feces were thawed to allow for mixing, and composited by period and dog. Samples were stored at −20°C for a period not longer than 3 months.

For IgA analysis, fecal samples were thawed, homogenized and subjected to saline extraction, according to the method described by Peters, Calvert (26), with modifications. Briefly, 1 g of fresh fecal sample was added to 10 mL of extraction buffer (PBS containing 0.5% Tween 20, Sigma-Aldrich, St. Louis, MO, USA), thoroughly homogenized using vortex and centrifuged at 1500 × g, 5°C, during 20 min. Then, 80 μL of a 25× concentrated solution of complete™ EDTA-free Protease Inhibitor Cocktail (04693132001, Roche, Basel, Switzerland) in PBS were added to 2 mL of supernatant, homogenized using vortex and centrifuged at 15000 × g, 5°C, during 15 min. The supernatants were then aliquoted and stored at −20°C until analysis. Fecal IgA concentrations were determined using a commercial ELISA kit (Dog IgA ELISA Quantitation Set, E44-104, Bethyl Laboratories Inc., Montgomery, TX, USA), according to the manufacturer’s instructions.

Samples were diluted 1:1000 or 1:2000 in dilution buffer, according to previous optimal dilution determination, and the analysis was carried out in duplicate. The colorimetric detection was performed using a Multiskan EX microplate reader (Thermo Fisher Scientific) using the Ascent software (Thermo Fisher Scientific).

For microbiome analysis, fecal DNA was extracted with E.Z.N.A.^®^ Stool DNA Kit (Omega Bio-tek, Inc., Norcross, GA, USA), following the supplier’s protocol. After DNA extraction, primers targeting the V4 region of the 16S rRNA gene (forward: GTGYCAGCMGCCGCGGTAA, reverse: GGACTACNVGGGTWTCTAAT) with attached adapters and barcodes were used for amplification. After amplification, samples were purified and sequenced on an Illumina Novaseq 6,000 sequencer. Raw reads were imported to Qiime2 (27). Primers/adapters were removed by the cutadapt (28). Denoising, quality filtering, merging of paired reads, and chimeras removal were done by the dada2 (29). Taxonomy classification of amplicon sequence variants (ASVs) was carried out by VSEARCH-based consensus (30) and pre-fitted sklearn-based classifiers (31) against the Silva database (v138.1, 16S 99%) (32). The reference reads were preprocessed by RESCRIPt (33). Alpha diversity was assessed by Shannon’s entropy (34) and Faith’s phylogenetic diversity (35) indices, and beta diversity by Weighted UniFrac (36) distances. Alpha diversity metrics were compared by the Wilcoxon test (37) for dependent samples, and beta diversity distances by the Adonis test (999 permutations) (38). Differentially abundant genera (only for counts of genera with relative abundance ≥1% and prevalence ≥20%) were detected by Linear discriminant analysis Effect Size (LEfSe) (39). *p*-values obtained from LEfSe were adjusted using the Benjamini-Hochberg procedure (40). Only features with p-adj > 0.05 and LDA > 2 were considered to be differentially abundant.

### 2.5 Statistical analysis

Data were analyzed using the mixed procedure of the Statistical Analysis Systems software package (SAS 2021, release 3.1.0., SAS Institute, Cary, NC, USA). The model included diet, period, and dietary sequence as fixed effects and animal within dietary sequence as random effect. The level of significance was set at *p* < 0.05, and a trend was considered for *p* < 0.10. Dietary sequence did not affect any measured parameters, except for serum insulin concentration and systolic arterial pressure.

## 3 Results

### 3.1 Clinical blood chemistry

The experimental diet decreased plasmatic triglycerides (*p* = 0.008) and the activity of angiotensin converting enzyme (*p* = 0.013), also tending to decrease total cholesterol (*p* = 0.062; Table 1). No diet effects were observed in the other blood parameters analyzed (Table 1).

**Table 1:** Effect of the control and experimental diets on clinical blood biochemistry.

	Diet^1^		SEM	*p*-value	Reference values
	Control	Experimental			
Total protein, g dL^−1^	5.94	6.02	0.079	0.122	5.5–7.2
Albumin, g dL^−1^	3.67	3.72	0.064	0.281	3.2–4.1
Globulin, g dL^−1^	2.27	2.30	0.039	0.510	1.9–3.7
Glucose, mg dL^−1^	99.4	102.5	1.82	0.229	76–119
Insulin, mU L^−1^	12.3	12.1	1.14	0.880	5–20
Creatinine, mg dL^−1^	0.945	0.952	0.0570	0.900	0.6–1.4
Urea, mg dL^−1^	29.4	28.2	1.98	0.378	19.3–55.6
Alanine aminotransferase, U L^−1^	32.6	32.5	5.86	0.966	17–95
Alkaline phosphatase, U L^−1^	28.1	29.7	3.94	0.201	7–115
Cholesterol, mg dL^−1^	138.5	134.2	5.73	0.062	135–278
Triglycerides, mg dL^−1^	34.6	31.7	1.92	0.008	23–102
Cholesterol HDL, mg dL^−1^	111.1	107.5	4.13	0.188	ND^2^
Cholesterol LDL, mg dL^−1^	<5.0	<5.0			ND
NT-pro-BNP, pmol L^−1^	1,008	1,030	120.3	0.819	<1800
Reactive C protein, mg L^−1^	5.33	8.85	1.848	0.189	0–12
Troponin I, ng mL^−1^	0.048	0.050	0.0050	0.731	0–0.7
Aldosterone, ng L^−1^	68.0	71.0	9.79	0.821	15–102
Renin activity, ng mL^−1^ h^−1^	1.43	1.51	0.164	0.756	0.4–1.9
Angiotensin II, pg mL^−1^	191.9	214.0	42.61	0.721	ND
Angiotensin converting enzyme activity, U L^−1^	32.5	28.0	3.95	0.013	ND
Insulin-like growth factor (IGF-1), μg L^−1^	144.5	150.1	12.82	0.730	105–280

^1^Extruded complete diet with inclusion of shrimp hydrolysate and salmon oil (control) or with fish hydrolysate and fish oil (experimental). Values presented as least square means. ^2^Not defined.

### 3.2 Serum cytokine and adipokine concentrations

Diet did not significantly affect serum levels of the pro-inflammatory cytokines IL-1β (*p* = 0.117), IL-8 (*p* = 0.960), and IL-12/IL-23 p40 (*p* = 0.332), and the serum levels of the anti-inflammatory adipokine adiponectin (*p* = 0.398; Table 2). Concentrations of IL-10, TNF-*α*, and IFN-*γ* were below the detection levels (31.2 pg. mL^−1^, 15.6 pg. mL^−1^, and 31.2 pg. mL^−1^, respectively) for most of the animals, regardless of the diet (data not shown). Moreover, serum levels of the pro-inflammatory adipokine leptin were also below detection level (0.3438 ng mL^−1^) for all dog samples analysed, although ELISA quality controls 1 and 2 were within the expected range (2.52 and 13.52 ng mL^−1^, respectively).

**Table 2:** Effect of the control and experimental diets on serum cytokine and adipokine concentrations.

	Diet^1^		SEM	*p*-value
	Control	Experimental		
IL-1β, pg mL^−1^	936.6	1025.7	286.1	0.117
IL-8, pg mL^−1^	674.5	690.6	235.6	0.960
IL-12/IL-23 p40, pg mL^−1^	999.9	1075.1	207.1	0.332
Adiponectin, μg mL^−1^	13.7	12.8	0.74	0.398

^1^Extruded complete diet with inclusion of shrimp hydrolysate and salmon oil (control) or with fish hydrolysate and fish oil (experimental). Values presented as least square means.

### 3.3 Blood pressure, electrocardiogram, and echocardiogram

The electrocardiographic tracing acquired during the echocardiographic examination revealed sinus rhythm in all dogs and experimental conditions. Blood pressure values and heart rate did not exhibit any difference between the control and experimental diets with the only exception of systolic arterial pressure that tended (*p* = 0.097) to be higher in dogs fed the experimental diet (Table 3).

**Table 3:** Effect of the control and experimental diets on heart rate, systolic, diastolic, and average arterial pressures.

	Diet^1^		SEM	*p*-value
	Control	Experimental		
Heart rate, bpm	79.3	80.5	3.60	0.823
Systolic arterial pressure, mmHg	125.0	137.2	4.70	0.097
Diastolic arterial pressure, mmHg	86.4	93.1	5.51	0.412
Average arterial pressure, mmHg	96.1	102.5	5.52	0.430

^1^Extruded complete diet with inclusion of shrimp hydrolysate and salmon oil (control) or with fish hydrolysate and fish oil (experimental). Values presented as least square means.

Regarding echocardiographic assessment, the LA/Ao ratio in dogs following the experimental diet showed a significant increase (*p* = 0.029) compared to the control diet, with no other significant differences being observed in the other echocardiographic measurements (Table 4).

**Table 4:** Effect of the control and experimental diets on echocardiogram parameters.^1^

	Diet^2^		SEM	*P*-value
	Control	Experimental		
IVSd, cm	0.896	0.860	0.0327	0.200
IVSs, cm	1.16	1.15	0.047	0.759
LVIDd, cm	3.25	3.26	0.082	0.879
LVIDs, cm	2.37	2.37	0.082	0.981
LVPWd, cm	0.832	0.837	0.0219	0.859
LVPWs, cm	1.08	1.09	0.041	0.901
EF, Teich, %	53.6	53.7	2.29	0.982
FS, %	27.0	27.1	1.47	0.993
LVIDdN	1.57	1.56	0.033	0.810
LA, cm	2.70	2.82	0.084	0.162
Ao, cm	1.82	1.77	0.035	0.004
LA/Ao	1.48	1.59	0.035	0.029
EPSS, cm	0.330	0.281	0.0269	0.167
PV, m/s	0.654	0.669	0.0254	0.554
PV, mmHg	1.76	1.82	0.140	0.629
AV, m/s	1.008	0.984	0.0615	0.756
AV, mmHg	4.29	4.02	0.510	0.689
MV E, m/s	0.603	0.585	0.0229	0.586
MV A, m/s	0.398	0.391	0.026	0.828
MV E/A	1.66	1.53	0.104	0.385
EDV, mL	42.6	43.3	2.64	0.759
ESV, mL	20.7	19.2	1.72	0.289
SV, mL	21.9	24.1	1.49	0.174
CO, L/min	1.68	1.84	0.168	0.425

^1^Ao, aorta; AV m/s, aortic peak systolic velocity; AV mmHg, aortic systolic pressure gradient (mmHg); CO, cardiac output; EDV, end-diastolic volume; ESV, end-systolic volume; EF, ejection fraction; EPSS, E-point to septal separation; FS, fractional shortening; IVSd, interventricular septum thickness in diastole; IVSs, interventricular septum thickness in systole; LA, left atrium; LA/Ao, left atrial to aorta diameter, short axis; LVIDd, left ventricular internal diameter in diastole; LVIDdN, left ventricular internal diameter in diastole normalized to body weight; LVIDs, left ventricular internal diameter in systole; LVPWd, left ventricular posterior wall thickness in diastole; LVPWs, left ventricular posterior wall thickness in systole; MV E m/s, mitral valve peak E wave velocity; MV A m/s, mitral valve peak A wave velocity; MV E/A, mitral valve E wave velocity to A wave velocity; PV m/s, pulmonary artery peak systolic velocity; PV mmHg, pulmonary artery systolic pressure gradient (mmHg); SV, stroke volume. ^2^Extruded complete diet with inclusion of shrimp hydrolysate and salmon oil (control) or with fish hydrolysate and fish oil (experimental). Values presented as least square means.

### 3.4 Fecal immunoglobulin A concentration and microbiota

Diet did not significantly affect fecal IgA concentrations that averaged 5.31 and 7.37 mg g^−1^ of dry feces (SEM = 1.496) for the control and experimental diet, respectively (*p* = 0.200).

**Figure 1:**
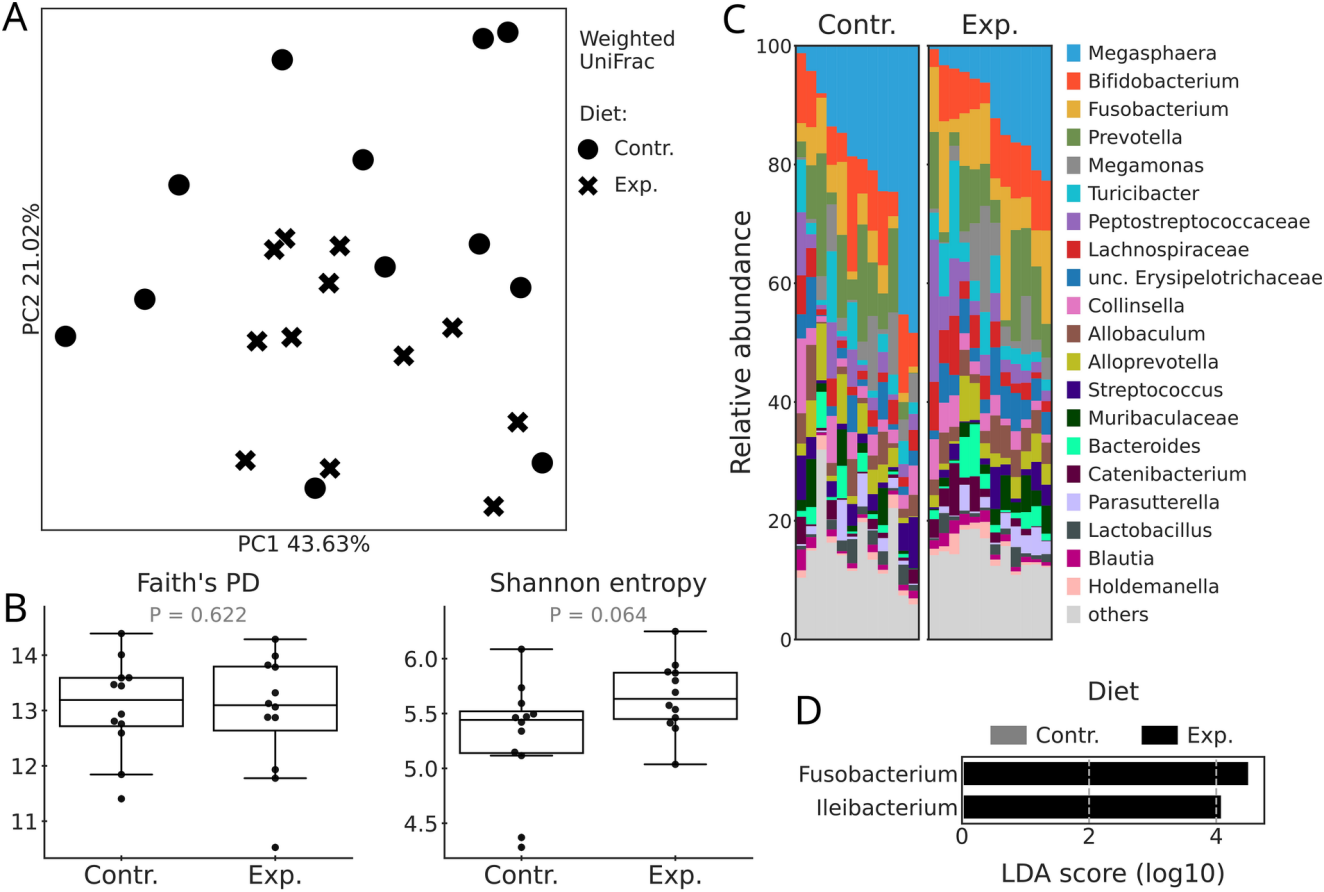
Microbiome diversity and composition of dogs fed with the control (Contr.) and experimental (Exp.) diets. (A) PCoA plots based on Weighted UniFrac distances. Diets are differentiated by the shape of the marker. (B) Boxplots based on Shannon entropy and Faith’s phylogenetic diversity. (C) Taxonomy barplots by diet at the genus level. If genus level was not assigned, the last available taxonomy rank was used for the label. (D) Differentially abundant genera between diets according to the LEfSe test (p-adj < 0.05, LDA > 2).

No significant differences (*p* = 0.155) in beta diversity (expressed by Weighted UniFrac) between the control and experimental diets were detected when tested with Adonis (Figure 1A). Similarly, diet (Figure 1B) did not significantly affect alpha diversity metrics (Shannon entropy and Faith PD) of dogs’ fecal microbiome, although the Shannon entropy had a tendency to be higher in the experimental diet (*p* = 0.064).

In both control and experimental diets, *Megasphaera* was the most abundant genus, followed by *Bifidobacterium*, *Fusobacterium*, and *Prevotella* (Figure 1C). A differential abundance test (LEfSe) showed that the relative abundances of only two genera, *Fusobacterium* and *Ileibacterium* were greater in the experimental diet (Figure 1D).

## 4 Discussion

Fish hydrolysate and oil obtained from the local agrifood sector contributes both for the problem of fish waste disposal and to the sustainability of the petfood sector. The present study evaluated the effects of the dietary replacement of shrimp hydrolysate and salmon oil with fish hydrolysate and oil on systemic inflammation markers, adipokine levels, cardiac function and fecal microbiota of adult dogs from a feeding trial earlier reported (14).

The clinical blood chemistry parameters evaluated were all within the normal ranges for healthy canines. The tendency for a decreased total cholesterol and a significant decrease of triglycerides observed with the experimental diet could be mainly attributed to its higher content of DHA and EPA as verified in previous studies (41, 42). Although not clearly understood, the mechanisms of action of omega-3 PUFA might be attributed, among others, to the regulation of transcription factors that reduce lipogenesis, increased ß-oxidation and stimulation of the activity of the lipoprotein lipase (43–45).

Leptin, a pro-inflammatory adipokine mainly secreted by adipose tissue and that positively correlates with body fat content (46, 47), was below detection level in all dog samples analysed, although ELISA quality controls were within the expected range. Similarly, it was shown by others using the same commercial ELISA kit used here that in dogs with ideal weight the levels of leptin were undetectable in the majority of the animals, in contrast with obese animals in which leptin levels were detectable (48). The levels of the adipokine adiponectin, that can have anti-inflammatory properties (49), were detectable in all animals within the range reported by others for healthy dogs (50). Decreased adiponectin serum levels in dogs have been associated with obesity (51, 52). During this study no changes in adiponectin levels were observed, which is in accordance with no detected changes in BW.

Protein hydrolysate and oil from fish by-products did not significantly influence basal levels of inflammation, which is in accordance with the similar levels of C-reactive protein detected in the serum of dogs under control and experimental diets. A high variability in the serum levels of the pro-inflammatory cytokines IL-1β, IL-8 and IL-12/IL-23 p40 was observed among the different dogs, regardless of diet, while IL-10, TNF-*α* and IFN-*γ* were below the detection limits for most animals. Previous research has reported variations in the levels of different cytokines among individuals, irrespective of breed, age and sex, being the serum levels of the cytokines IL-10, TNF-α and IFN-γ usually reported to be low or undetectable (53–56). Increased serum levels of IL-8 and IL-1β have been associated with both acute and chronic inflammation originated from diverse pathological conditions (53, 57–59), including heart disease (52, 60). The lack of differences in the levels of these particular cytokines in the present study are in line with the absence of significant alterations in hematological and cardiac parameters.

Hydrolysed proteins have been used in diets for dogs with adverse food reactions or gastrointestinal disorders (61). In several studies, the inclusion of protein hydrolysates did not significantly affect serum or plasma cytokine levels (62, 63). However, inclusion of black soldier fly larvae and microalgae-like *Schizochytrium* protein hydrolysates in the diet decreased serum IL-8, suggesting a potential anti-inflammatory role for these particular hydrolysates in dogs (64). Discrepant reported results on dietary inclusion of protein hydrolysates in dogs may be explained by many variables, including the hydrolysate origin, the percentage of inclusion and the duration of the feeding trial.

In the present study, both diets were fed to healthy dogs and contain oil from fish sources, rich in EPA and DHA (14). The role of EPA and DHA in inflammation is pleiotropic by influencing several biological processes, such as cell membrane composition and fluidity, production of prostaglandins E (PGE), leukotrienes B (LTB), resolvins and protectins, and signaling pathways involved in the expression of pro-inflammatory genes (65, 66). The replacement of shrimp hydrolysate and salmon oil by fish hydrolysate and oil led to an increase of EPA and DHA contents resulting in significantly higher concentrations of these PUFA in the red blood cells of dogs fed the experimental diet (2.02 vs. 1.41 g 100 g^−1^ of EPA and 1.75 vs. 1.17 g 100 g^−1^ of DHA for experimental and control diet, respectively) (14). In a large human community-based cohort, the EPA and DHA concentrations of red blood cells were inversely correlated with several markers of inflammation (67). In that line, several authors have suggested that EPA and DHA-enriched diets could be used as part of anti-inflammatory treatments in dogs with chronic inflammatory diseases. As such, the introduction of fish oil in diets for dogs of different breeds led to a decrease in the cellular production of the pro-inflammatory mediators PGE_2_ or LTB_4_ (68, 69) and in decreased production of IL-1β by mononuclear blood cells (6). The present study shows that shrimp hydrolysate and salmon oil can be replaced by protein hydrolysate and oil from fish by-products without affecting systemic inflammatory markers. Still, as differences were observed in EPA and DHA content of red blood cells of dogs fed the experimental diet, further studies using longer periods of dietary supplementation are necessary to assess long-term effects of these food resources.

The present study demonstrates that an experimental diet containing fish hydrolysate and oil has no significant impact in most of the parameters related to cardiac structure and function, as indicated by the results obtained from cardiac biomarkers (NT-proBNP and troponin I), blood pressure, and echocardiographic assessments; also, it is important to refer that all parameters felt within the reference range for a healthy adult dog (20, 70, 71). While a significant increase in the echocardiographic LA/Ao ratio, a marker of left atrial enlargement, was observed in the experimental diet group, all values remained within the reference range for dogs (72). These findings suggest that the diet under investigation does not impact cardiac remodeling; however, it is important to note that the assessment was conducted over a short duration. Therefore, it is crucial to evaluate the diet’s long-term effects on cardiac health. In myxomatous mitral valve disease, the most prevalent naturally occurring heart condition in dogs, left atrial enlargement serves as the most reliable independent indicator of cardiac disease progression. This disease is associated with changes in energy metabolism, oxidative stress, and inflammation. Interestingly, a prior study demonstrated that a diet supplemented with a blend including medium-chain triglycerides, EPA and DHA, magnesium, taurine and vitamin E, reduced systolic arterial blood pressure and left atrial enlargement in dogs with early preclinical myxomatous mitral valve disease (stage B2 and C), attributed to various properties, including its antioxidant and anti-inflammatory effects (73). Considering the antioxidant properties of fish hydrolysate and oil, it would be worthwhile in the future to assess its role in advanced stages of heart disease, with secondary cardiac remodeling and/or heart failure. Lower levels of serum adiponectin have been reported in dogs with valve disease (74, 75), in accordance with studies in mice and humans that show a role of this adipokine in the cardiovascular system (76, 77). According to no major changes observed in cardiac parameters we did not observe significantly changes in the adiponectin serum levels in animals fed the experimental diet.

In addition, the experimental diet decreased the angiotensin converting enzyme activity, suggesting a potential role of this diet on mitigating the renin-angiotensin-aldosterone system, a neurohormonal system that function to preserve intravascular volume and perfusion pressure in situations of decreased cardiac output (78). Moreover, considering the recommended dose of 40 mg kg^−1^ BW of EPA and 25 mg kg^−1^ DHA to achieve a cardioprotective effect (6), the intake of EPA and particularly of DHA with the experimental diet (18 mg kg^−1^ BW of EPA and 28 mg kg^−1^ BW of DHA) might benefit the cardiac function of dogs.

The experimental diet containing fish hydrolysate and oil had no significant effect on the fecal IgA and the composition of the canine fecal microbiome compared to a commonly used commercial diet including shrimp hydrolysate and salmon oil. Secretory IgA, the most abundant immunoglobulin in mucous secretions, plays a significant role in mucosal immunity and promotes gut health (79). Gut secretory IgA is an important modulator of gut microbiota (80), and the reverse also occurs, being well documented in how gut microbiota drives IgA production (81, 82). Only two genera, *Fusobacterium* and *Ileibacterium* increased their relative abundance when the experimental diet was applied. Care should be taken in driving strong conclusions of these results as the authors are aware of the limitations of using LEfSe in such analysis (83). Being *Fusobacterium* a butyrate producer that utilizes lysine degradation pathways to produce butyrate from protein sources, an increase in its abundance has been reported in dogs fed high protein diets (84–86). However, in the present study, the experimental diet presented slightly lower protein and lysine content than the control diet, with no differences being observed on butyrate concentration among diets (14). *Fusobacterium*, despite being associated with inflammatory bowel disease (87) and colorectal cancer (88) in humans, is attributed to the healthy microbiome in dogs (89). Not much information is available on the role that *Ileibacterium* plays in dogs’ health but in mice, this genus was associated with reduced risks of inflammation and dysbiosis (90, 91). The results demonstrate that the diet containing fish hydrolysate and oil can be used as the substitution for shrimp hydrolysate without drastic changes in dogs’ fecal microbiome, while potentially propagating bacterial genera associated with the healthy canine microbiome. It is known that the preprocessing storage temperatures may affect the gut microbiome composition in dogs (92). However, despite the samples in this study were not stored at the optimal temperature, they follow optimized standardized protocols from sample collection, storage and processing, providing that all samples were treated in the same conditions, the findings were not affected by it. However, additional metabolomic and metagenomics analysis could be useful in elucidating the relevance and role of these microbiota changes in the gut.

## 5 Conclusion

The present short term study shows that replacing imported shrimp hydrolysate and salmon oil with locally produced fish oil and hydrolysate from the agrifood industry did not affect systemic inflammatory markers, cardiac structure and function, but potentially benefit bacterial genera associated with healthy canine microbiome. Future studies should address the long-term effects of these food resources and their role in spontaneous canine cardiac diseases.

## Data Availability

The datasets presented in this study can be found in online repositories. The names of the repository/repositories and accession number(s) can be found at: https://www.ebi.ac.uk/ena, PRJEB74498.
